# Bleeding prevention with desmopressin for allograft kidney biopsies: a double-blind randomized study

**DOI:** 10.1093/ckj/sfag083

**Published:** 2026-03-12

**Authors:** Gabriel Sartori Pacini, Arthur Gus Manfro, Renata Asnis Schuchmann, Vanderlei Carlos Bertuol Junior, Amanda Vilaverde Perez, Eduarda Taís Schneider, Leonardo Luigi Adams Backes, Gabriela Vieira Steckert, Roberto Ceratti Manfro, Andrea Carla Bauer

**Affiliations:** Division of Nephrology, Hospital de Clínicas de Porto Alegre, Rua Ramiro Barcelos 2350, Porto Alegre, Rio Grande do Sul, Brazil; Postgraduate Program in Medical Sciences, Endocrinology, Universidade Federal do Rio Grande do Sul, R. Ramiro Barcelos 2400, Porto Alegre, Brazil; Division of Nephrology, Hospital de Clínicas de Porto Alegre, Rua Ramiro Barcelos 2350, Porto Alegre, Rio Grande do Sul, Brazil; Postgraduate Program in Medical Sciences, Endocrinology, Universidade Federal do Rio Grande do Sul, R. Ramiro Barcelos 2400, Porto Alegre, Brazil; Division of Transplantation, Hospital de Clínicas de Porto Alegre, Porto Alegre, Rio Grande do Sul, Brazil; Division of Nephrology, Hospital de Clínicas de Porto Alegre, Rua Ramiro Barcelos 2350, Porto Alegre, Rio Grande do Sul, Brazil; Division of Transplantation, Hospital de Clínicas de Porto Alegre, Porto Alegre, Rio Grande do Sul, Brazil; Postgraduate Program in Medicine, Medical Sciences, Universidade Federal do Rio Grande do Sul, Porto Alegre, Brazil; Division of Nephrology, Hospital de Clínicas de Porto Alegre, Rua Ramiro Barcelos 2350, Porto Alegre, Rio Grande do Sul, Brazil; Postgraduate Program in Medical Sciences, Endocrinology, Universidade Federal do Rio Grande do Sul, R. Ramiro Barcelos 2400, Porto Alegre, Brazil; Division of Transplantation, Hospital de Clínicas de Porto Alegre, Porto Alegre, Rio Grande do Sul, Brazil; Postgraduate Program in Medical Sciences, Endocrinology, Universidade Federal do Rio Grande do Sul, R. Ramiro Barcelos 2400, Porto Alegre, Brazil; Division of Nephrology, Hospital de Clínicas de Porto Alegre, Rua Ramiro Barcelos 2350, Porto Alegre, Rio Grande do Sul, Brazil; Division of Nephrology, Hospital de Clínicas de Porto Alegre, Rua Ramiro Barcelos 2350, Porto Alegre, Rio Grande do Sul, Brazil; Division of Nephrology, Hospital de Clínicas de Porto Alegre, Rua Ramiro Barcelos 2350, Porto Alegre, Rio Grande do Sul, Brazil; Division of Transplantation, Hospital de Clínicas de Porto Alegre, Porto Alegre, Rio Grande do Sul, Brazil; Division of Nephrology, Hospital de Clínicas de Porto Alegre, Rua Ramiro Barcelos 2350, Porto Alegre, Rio Grande do Sul, Brazil; Division of Transplantation, Hospital de Clínicas de Porto Alegre, Porto Alegre, Rio Grande do Sul, Brazil; Postgraduate Program in Medicine, Medical Sciences, Universidade Federal do Rio Grande do Sul, Porto Alegre, Brazil; Department of Internal Medicine, School of Medicine - Universidade Federal do Rio Grande do Sul, Porto Alegre, Brazil; Division of Nephrology, Hospital de Clínicas de Porto Alegre, Rua Ramiro Barcelos 2350, Porto Alegre, Rio Grande do Sul, Brazil; Postgraduate Program in Medical Sciences, Endocrinology, Universidade Federal do Rio Grande do Sul, R. Ramiro Barcelos 2400, Porto Alegre, Brazil; Division of Transplantation, Hospital de Clínicas de Porto Alegre, Porto Alegre, Rio Grande do Sul, Brazil; Department of Internal Medicine, School of Medicine - Universidade Federal do Rio Grande do Sul, Porto Alegre, Brazil

**Keywords:** bleeding, desmopressin, kidney biopsy, kidney transplantation, renal biopsy

## Abstract

**Background:**

Percutaneous kidney allograft biopsy is essential for evaluating graft dysfunction, but post-procedural bleeding remains the most frequent complication, particularly among recipients with impaired renal function. Desmopressin (DDAVP) is often used prophylactically to reduce bleeding risk, yet its efficacy in the transplant setting remains uncertain.

**Methods:**

We conducted a single-center, double-blind, randomized, placebo-controlled trial at a quaternary transplant center in Brazil. Adult kidney transplant recipients with estimated glomerular filtration rate (eGFR) <60 ml/min/1.73 m² undergoing allograft biopsy were randomized (1:1) to receive intravenous desmopressin (0.3 μg/kg) or placebo before biopsy. All procedures were ultrasound-guided and performed by experienced nephrologists. The primary endpoint was any biopsy-related bleeding complication. Secondary outcomes included major bleeding (transfusion, embolization, nephrectomy, or death), minor bleeding (macroscopic hematuria, hematoma, hemoglobin drop >20%), and hyponatremia.

**Results:**

A total of 96 biopsies were randomized (48 per group). Baseline demographics, clinical, and procedural characteristics were well balanced. Any biopsy-related bleeding complication occurred in 29.1% of procedures overall, without a significant difference between the desmopressin and placebo groups (35.4% vs. 22.9%, *P* = .262). One major bleeding event occurred in the desmopressin group (2.1%) and none in the placebo group (*P* = 1.00). Minor bleeding was more frequent with desmopressin-treated patients (35.4% vs. 22.9%, *P* = .262) than in placebo, but without statistical significance. In adjusted analyses using penalized logistic regression, desmopressin was not associated with reduced bleeding risk [OR 1.61 (95%CI 0.63–4.20)]. By contrast, surveillance biopsies were independently associated with a markedly higher risk of minor bleeding compared with indication biopsies (adjusted OR 10.2; 95%CI 1.8–68.7). No cases of hyponatremia were documented, and adverse events were rare and balanced across groups.

**Conclusions:**

In this trial of high-risk kidney transplant recipients, prophylactic desmopressin did not reduce biopsy-related bleeding complications compared with placebo. Routine pre-biopsy administration is not supported when biopsies are performed under optimal technical conditions and patient preparation.

## Graphical Abstract

**Graphical Abstract:**
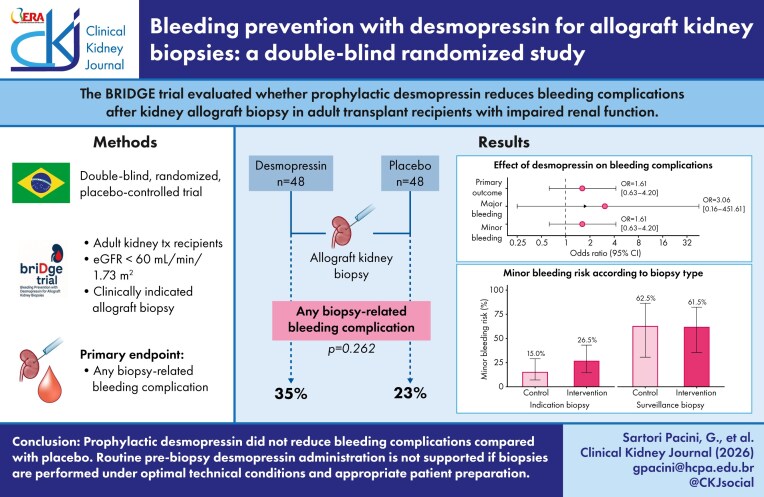

KEY LEARNING POINTS **What was known:**

- Percutaneous kidney biopsy is a crucial diagnostic tool for evaluating kidney allograft dysfunction.
- Bleeding remains the most important complication associated with the procedure.
- Evidence supporting the prophylactic use of desmopressin to reduce biopsy-related bleeding is inconsistent and largely derived from heterogeneous populations.

**This study adds:**

- This study is a double-blind randomized controlled trial evaluating prophylactic desmopressin in kidney allograft biopsy.
- Prophylactic desmopressin did not reduce biopsy-related bleeding complications compared with placebo in high-risk kidney transplant recipients.
- No clinical benefit was observed across predefined subgroups when biopsies were performed under optimized procedural conditions.

**Potential impact:**

- These findings suggest that routine prophylactic use of desmopressin before kidney allograft biopsy may warrant re-evaluation, particularly when biopsies are performed under optimized procedural conditions.
- Alternative approaches to hemostasis should be considered, potentially leading to the exploration of other treatments that may be more effective in managing bleeding complications.

## INTRODUCTION

Percutaneous kidney biopsy remains an essential diagnostic tool for evaluating allograft dysfunction in kidney transplant recipients. Despite advances in imaging and biopsy techniques, needle design, and procedural standardization, post-procedural bleeding continues to represent the most frequent complication, particularly in patients with impaired renal function. In this population, uremia-associated platelet dysfunction and the burden of comorbidities further increase bleeding risk [1–3].

Desmopressin (1-deamino-8-D-arginine vasopressin, DDAVP), a selective vasopressin V2-receptor agonist, promotes the release of von Willebrand factor and factor VIII, thereby enhancing platelet adhesion [3]. Although its prophylactic use before kidney biopsy is common in patients with increased bleeding risk, evidence supporting its efficacy remains limited and inconsistent [4–8]. While some randomized trials have reported reductions in minor bleeding events, others have failed to demonstrate any meaningful benefit, particularly regarding major bleeding [4–8].

In the transplant setting, biopsy-related bleeding carries not only immediate clinical consequences such as transfusion, invasive intervention, or graft loss, but also the potential long-term immunologic risks, including risk of allosensitization and may adversely affect long-term graft survival [9, 10]. Given the paucity of robust evidence supporting DDAVP’s efficacy in renal allograft biopsy and the heterogeneity in clinical practice, there is a need for rigorous evaluation of DDAVP in this setting.

We conducted a double-blind, randomized, placebo-controlled trial to evaluate the efficacy and safety of desmopressin for reducing bleeding complications following percutaneous allograft kidney biopsy in high-risk adult transplant recipients.

## MATERIALS AND METHODS

### Trial design

This was a prospective, single-center, double-blind, randomized, placebo-controlled clinical trial conducted at a quaternary university hospital in southern Brazil. Adult kidney transplant recipients undergoing a clinically indicated percutaneous allograft kidney biopsy during the study period (September 2024 to August 2025) were eligible for inclusion, irrespective of time since transplantation. The study was conducted in accordance with the Declaration of Helsinki and Good Clinical Practice guidelines and was approved by the institutional ethics committee. Written informed consent was obtained from all participants. The study was prospectively registered at ClinicalTrials.gov (Identifier: NCT06622187, registered in August 2024).

All biopsies were clinically indicated. Biopsies categorized as indication biopsies were performed in the setting of overt graft dysfunction, including worsening renal function, proteinuria, or the development of donor-specific antibodies, with the aim of establishing a clinicopathological diagnosis and guiding therapeutic decisions [11]. Biopsies referred to as surveillance biopsies were also clinically indicated and were performed in patients with delayed graft function, with the objective of enabling early detection of pathological processes other than acute tubular necrosis, such as cellular or antibody-mediated rejection [12].

### Participants

Eligible participants were adult kidney transplant recipients (≥18 years) with an estimated glomerular filtration rate (eGFR) <60 ml/min/1.73 m² who were scheduled for a clinically indicated allograft biopsy. Inclusion criteria required a platelet count ≥80 000/µl, normal prothrombin time and activated partial thromboplastin time, and systolic blood pressure <160 mmHg within 24 hours of the procedure. Exclusion criteria included pregnancy, hypersensitivity to desmopressin, use of therapeutic anticoagulation or dual antiplatelet therapy, and a history of bleeding disorders such as type IIB von Willebrand disease, and a history of clinically significant bleeding following a previous kidney biopsy.

### Randomization and blinding

Participants were randomly assigned in a 1:1 ratio to receive either intravenous desmopressin (0.3 μg/kg diluted in 100 ml of 0.9% saline) or 100 ml of 0.9% saline alone (placebo). Randomization was performed using a computer-generated simple randomization sequence without blocking or stratification. The allocation sequence was managed by an independent study coordinator who was not involved in patient care or outcome assessment.

To maintain allocation concealment, each participant received a unique identifier linked to the assigned treatment group in a secure database accessible only to the coordinator. Study medications were prepared in identical 100 ml saline bags to ensure visual indistinguishability. The intravenous route was chosen to ensure predictable bioavailability, rapid onset of action, and precise weight-based dosing, and has been previously used in studies evaluating desmopressin in the context of kidney biopsy and surgical bleeding [13, 14].

Study infusions were prepared by nursing staff who were unblinded to treatment allocation. Infusion administration and post-procedure patient monitoring were performed by blinded nursing staff, using identical infusion bags to ensure allocation concealment. Treating physicians, investigators, and outcome assessors remained blinded throughout the study.

### Procedure

Allograft kidney biopsies were performed in either inpatient or outpatient settings based on clinical indications. Procedures were conducted by one of two senior nephrologists with extensive experience in percutaneous renal biopsy, using real-time ultrasound guidance (Mindray MX7 systems, Mindray Medical International, Shenzhen, China). Patients were positioned supine, and biopsies were performed using a 16-gauge automatic spring-loaded biopsy needle. A minimum of two cortical tissue cores were obtained per biopsy; additional passes were performed only when required to ensure diagnostic adequacy.

Participants in the Intervention group received desmopressin at a dose of 0.3 μg/kg diluted in 100 mL of 0.9% saline and administered intravenously over 20 minutes, ∼1 hour before the biopsy. The Control group received an identical volume of 0.9% saline administered in the same protocol. Infusions were prepared and administered by trained nursing staff who were unblinded to treatment assignment; all other study personnel, including physicians and outcome assessors, remained blinded. Peri-procedural fluid administration was controlled, and post-biopsy monitoring was standardized across all participants.

Following the biopsy, all patients remained on strict bed rest for 8 hours and were kept under clinical observation for at least 24 hours, in accordance with institutional protocol. Standardized post-procedure monitoring was applied to all participants. The use of additional hemostatic agents, including tranexamic acid or vitamin K, was not permitted per protocol and was not administered to any participant to avoid confounding effects. Post-biopsy monitoring included vital sign surveillance, serial laboratory measurements, and renal ultrasound between 8 and 24 hours after the biopsy to screen for hematoma formation, regardless of symptoms. Additional imaging and clinical management were performed as needed, per institutional protocol.

### Outcomes

The primary outcome was the occurrence of any biopsy-related bleeding complication, including both major and minor bleeding events. Minor bleeding was defined as macroscopic hematuria, ultrasound-detected hematoma, and/or a hemoglobin drop >20%. Major bleeding was defined as bleeding requiring blood transfusion, embolization, graft nephrectomy, or resulting in procedure-related death. Bleeding events were assessed within 48 hours after the biopsy, in accordance with the study protocol.

### Statistical analyses

All analyses were performed using R (R Foundation for Statistical Computing, Vienna, Austria). Continuous variables are presented as mean ± standard deviation, and categorical variables as counts and percentages. Group comparisons were conducted using Fisher’s exact test or Pearson’s chi-squared test, as appropriate. Because major bleeding events were rare, Fisher’s test was used for that outcome.

To address low event rates and potential data separation, adjusted analyses of binary outcomes were performed using Firth’s penalized logistic regression (logistf package) was applied to estimate adjusted odds ratios (ORs) with 95% confidence intervals (CIs). Multivariable adjustment for major bleeding was not attempted due to a single event. For minor bleeding, the model included a treatment group with adjustments for biopsy indication, AKI, and eGFR. Model complexity was limited given the number of events. Effect modification by AKI was explored using an interaction term (group × AKI). When zero-cell counts occurred, Haldane–Anscombe corrected ORs (continuity correction of 0.5) were reported descriptively.

Sensitivity analysis incorporated aspirin use or pre-biopsy mean arterial pressure into the minor bleeding model. For descriptive analyses, histopathological diagnoses from kidney allograft biopsies were grouped into clinically relevant categories [acute tubular necrosis (ATN)/no major findings, rejection, glomerular disease, or other diagnoses] to improve interpretability and avoid sparse data. All statistical tests were two-sided, and a *P* < .05 was considered statistically significant.

### Sample size

The sample size was determined a priori based on previously published data. Assuming a two-sided *α* of 0.05, 80% statistical power, and an expected incidence of overall bleeding of 55% in the Control group versus 23.3% in the desmopressin group—values derived from Sattari *et al*. [8]—we calculated that 86 patients (43 per group) would be required to detect this absolute risk reduction. Accounting for an anticipated 10% attrition rate, the final target sample size was set at 96 patients. This calculation was designed to detect differences in overall bleeding; however, the study was not powered to detect differences in major bleeding events, which are rare in transplant kidney biopsies (<1%–2% in contemporary series).

## RESULTS

### Trial participants

**Figure 1:**
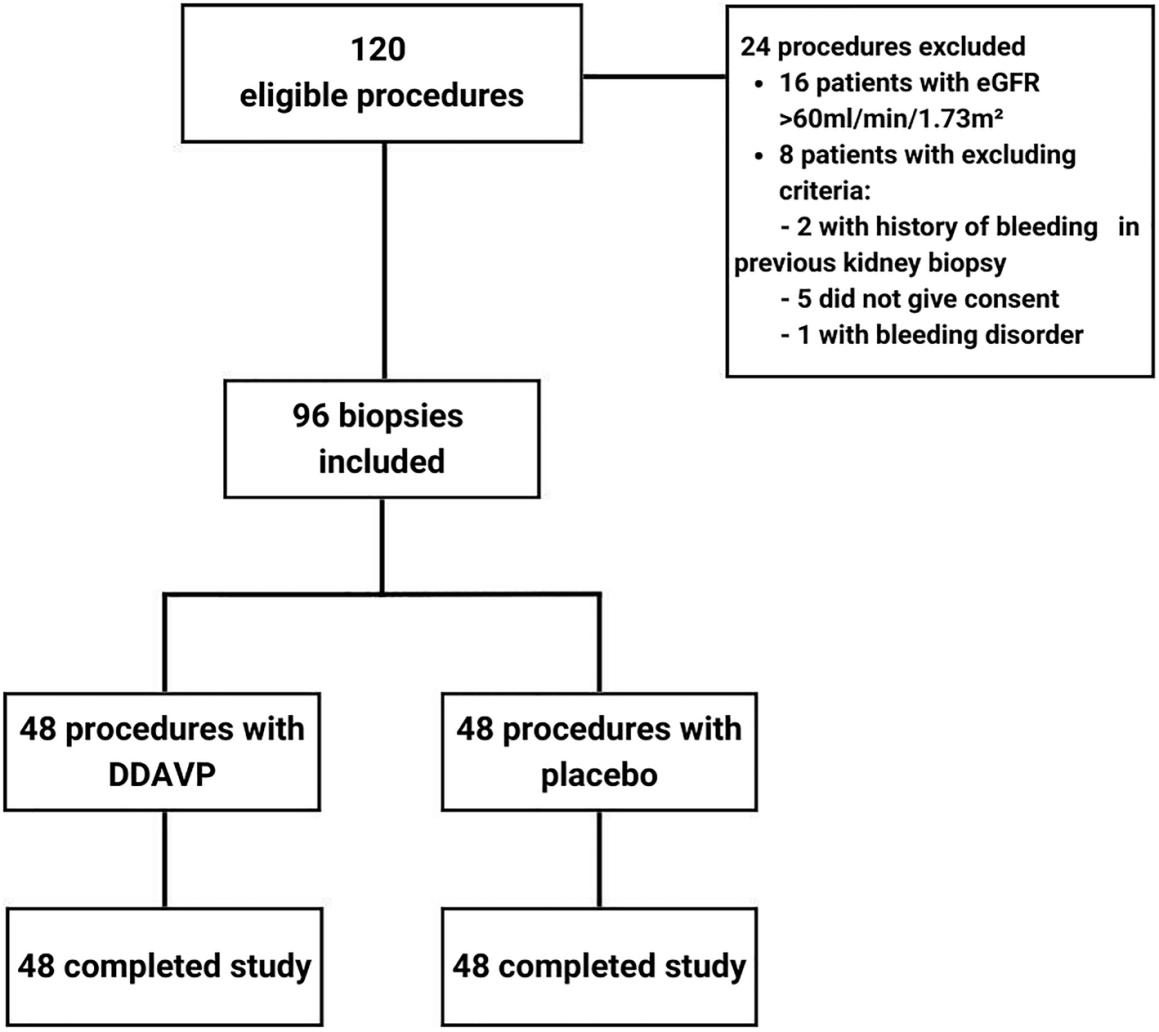
Flow diagram of patient selection and randomization.

Between September 2024 and August 2025, 120 kidney transplant biopsies were screened. Sixteen were excluded at screening for preserved renal function, not meeting the inclusion criterion of eGFR <60 ml/min/1.73 m². Of 104 eligible, five declined to provide informed consent, two had prior biopsy-related bleeding, and one had a documented bleeding diathesis. A total of 96 biopsies were therefore randomized and underwent the allograft biopsy procedure. The study flow diagram is presented in Fig. 1.

Baseline demographic, clinical, and laboratory characteristics were well balanced between groups (Table 1). The mean age was 53.0 ± 14.3 years in the desmopressin group and 49.4 ± 14.9 years in the placebo group. Female gender was more common in the Control group (54.2% vs. 43.8%). The distribution of race, comorbidities, and underlying kidney disease was similar between groups. Hypertension was the leading comorbidity (83% vs. 79%), followed by diabetes (32% vs. 26%). Table 1 describes the study population characteristics.

**Table 1:** Baseline demographic, clinical, laboratory, and biopsy characteristics of the study population, according to the treatment group.

Variable	Control(*n* = 48)	Intervention(*n* = 48)
Sex (female, %)	26 (54.2)	21 (43.8)
Age (years)	49.4 ± 14.9	53.0 ± 14.3
Aspirin (yes, %)	8 (16.%)	7 (14.6)
BMI (kg/m²)	27.1 ± 4.7	27.3 ± 4.3
eGFR (ml/min/1.73 m²)	22.6 ± 13.0	20.9 ± 13.2
Post-transplant time (months)	30.0 ± 51.4	28.9 ± 54.2
AKI (%)	31 (64.6)	33 (68.8)
Biopsy indication		
Indication (%)	40 (83.3)	34 (72.3)
Surveillance (%)	8 (16.7)	13 (27.7)
Pre-biopsy dialysis (%)	9 (18.8)	14 (29.2)
Pre-biopsy MAP (mmHg)	91.8 ± 10.2	92.6 ± 12.2
Pre-biopsy hemoglobin (g/dl)	9.9 ± 2.4	10.2 ± 2.5
Post-biopsy hemoglobin (g/dl)	9.8 ± 2.4	9.7 ± 2.3
Pre-biopsy sodium (mEq/l)	139.6 ± 2.7	138.4 ± 3.6
Post-biopsy sodium (mEq/l)	139.4 ± 2.9	138.6 ± 4.1
Platelet count (×10^9^/l)	212.6 ± 65.8	226.0 ± 73.8
INR	1.1 ± 0.1	1.0 ± 0.1
aPTT (seconds)	29.5 ± 3.4	28.4 ± 3.1
Biopsy diagnosis (grouped)		
ATN*/no major findings (%)	28 (58.3)	20 (41.7)
Rejection (%)	8 (16.7)	12 (25.0)
Glomerular disease (%)	5 (10.4)	8 (16.7)
Other diagnoses (%)	7 (14.6)	8 (16.7)

Kidney function was similarly reduced in both groups (20.9 ± 13.2 vs. 22.6 ± 13.0 ml/min/1.73 m²). There were no significant differences in baseline blood pressure, hemoglobin levels, platelet counts, or coagulation tests. Biopsy-related features, including the proportion of indication versus surveillance biopsies, and adequacy of specimens, were balanced across groups. Histopathological diagnoses were grouped into clinically meaningful categories for descriptive purposes, including tubular injury/no major findings, rejection, glomerular disease, and other diagnoses. The distribution of these diagnostic categories was similar across groups, with acute tubular necrosis (ATN)/no major findings being the most frequent finding, followed by rejection and glomerular disease (Table 1).

Biopsy adequacy was high and comparable between groups, with most procedures yielding diagnostically adequate cortical tissue.

### Outcomes

#### Primary outcome

Any biopsy-related bleeding complication occurred in 28 of 96 procedures (29.1%) of the procedure overall, with no statistically significant difference between groups. Bleeding events were documented in 17 of 48 patients (35.4%) in the desmopressin group and in 11 of 48 (22.9%) in the placebo group (*P* = .262).

**Figure 2:**
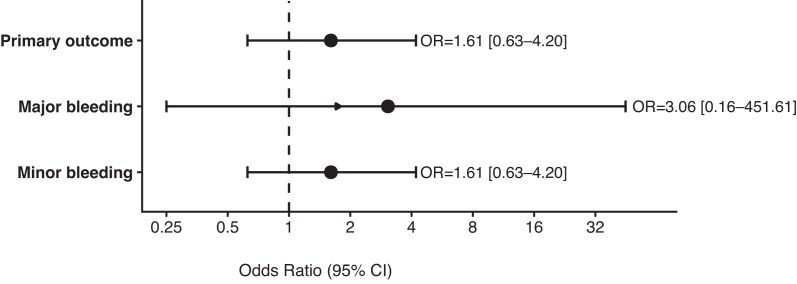
Effect of DDAVP intervention on bleeding complications. Bleeding-related variables by treatment group. ORs with 95% CIs are shown for the primary outcome, major bleeding, and minor bleeding.

Given the low number of bleeding events, adjusted analyses were performed using Firth’s penalized logistic regression. Multivariable modeling for major bleeding was not performed due to a single event. For minor bleeding, the prespecified primary adjusted model included treatment group (desmopressin vs placebo), biopsy indication (surveillance vs indication) and AKI (Table 3a). In this model, desmopressin was not associated with reduced odds of minor bleeding compared with placebo (adjusted OR 1.61; 95% CI 0.63–4.20; *P* = .327) (Fig. 2).

**Figure 3:**
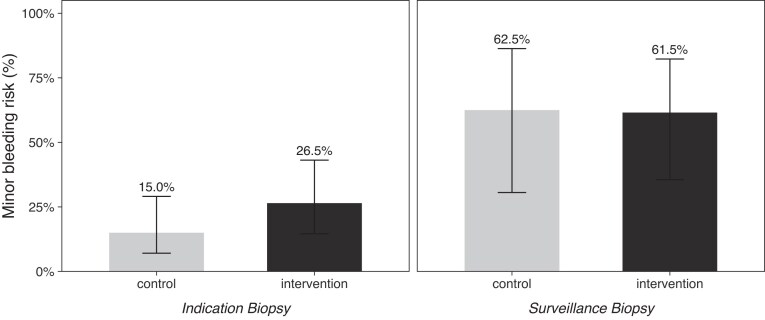
Minor bleeding risk according to biopsy type and treatment group. The proportion of minor bleeding events is shown for indication and surveillance kidney biopsies in the Control and Intervention (desmopressin) groups.

An interaction model including treatment group and biopsy indication was constructed to explore effect modification (Table 3b). Surveillance biopsies were independently associated with higher odds compared with indication biopsies (adjusted OR 10.18; 95% CI 1.79–68.70; *P* = .009). No significant interaction between treatment group and biopsy indication was observed (*P* for interaction = .524), indicating that the effect of desmopressin did not differ by biopsy indication (Fig. 3).

### Secondary outcomes

#### Minor bleeding

Minor bleeding occurred in 11/48 patients (22.9%) in the Control group and 17/48 (35.4%) in the Intervention group (*P* = .262) (Table 2). Ultrasound-detected hematomas were the most frequent manifestation in the Control and Intervention groups (21% vs. 31%), followed by macroscopic hematuria (2% vs. 10%) and arteriovenous fistula formation (2% vs. 6%) (Table 2). Some patients experienced more than one bleeding manifestation, therefore the total number of events exceeds the number of patients with bleeding complications.

**Table 2:** Bleeding-related variables by treatment group.

Variable	Control (*n* = 48)	Intervention (*n* = 48)	*P*
Δ Hemoglobin (g/dl)	−0.10 ± 0.57	−0.55 ± 0.85	.004
Δ Sodium (mEq/l)	−0.25 ± 1.78	0.12 ± 2.45	.393
Hematoma (%)	10 (20.8)	15 (31.2)	.352
Macroscopic hematuria (%)	1 (2.1)	5 (10.4)	.204
AVF (%)	1 (2.1)	3 (6.2)	.617
Blood transfusion needed (%)	0 (0.0)	0 (0.0)	1.000
Infection in hematoma (%)	0 (0.0)	0 (0.0)	1.000
Embolization (%)	0 (0.0)	1 (2.1)	1.000
Graftectomy (%)	0 (0.0)	0 (0.0)	1.000

In the primary adjusted Firth model, treatment assignment was not associated with minor bleeding (Table 3a). Biopsy indication emerged as the strongest predictor of bleeding risk. In an interaction model including treatment group and biopsy indication (Table 3b), surveillance biopsies were independently associated with higher odds of minor bleeding (adjusted OR 10.18; 95% CI 1.79–68.70; *P* = .009), whereas no evidence of effect modification by desmopressin was observed (*P* for interaction = .524). These findings indicate that the lack of treatment effect was consistent across biopsy indications.

**Table 3:** Regression models.

Model	Term	OR (95% CI)	*P* value
Table 3a. Treatment effect on bleeding outcomes (Firth penalized logistic regression).			
Major bleeding (unadjusted; Firth)	Intervention vs Control	3.06 (0.16–451.61)	.465
Minor bleeding (primary adjusted; Firth)	Intervention vs Control	1.61 (0.62–4.23)	.327
Table 3b. Effect modification by biopsy indication (interaction model for minor bleeding).			
Model	Term	OR (95% CI)	*P* value
Minor bleeding	Intervention vs Control	1.96 (0.64–6.36)	.242
Minor bleeding	surveillance vs indication	10.18 (1.79–68.70)	.009
Minor bleeding	interaction: (intervention × surveillance)	0.51 (0.06–4.03)	.524
Minor bleeding	AKI (yes vs no)	3.72 (0.94–17.92)	.062
Minor bleeding	eGFR (per 1 ml/min/1.73 m²)	1.04 (0.99–1.11)	.129
Minor bleeding	platelet count (per 1 × 10^9^/l)	1.00 (0.99–1.01)	.912

### Major bleeding

There was one major bleeding event overall [0/48 (0.0%) in Control vs. 1/48 (2.1%) in Intervention; Fisher’s exact *P* = 1.00]. The single major bleeding event required arteriovenous fistula embolization. No patients needed blood transfusions on graft nephrectomy, and there were no procedure-related deaths. Given the very low number of major bleeds, no multivariable modeling was performed for that endpoint.

### Sensitivity analyses

Adding aspirin use or pre-biopsy MAP to the primary adjusted model did not meaningfully change the treatment effect estimate (≤±3% relative change); and the overall conclusion remained unchanged (non-significant treatment effect) (Table 3a).

### Safety

Adverse events potentially related to desmopressin were rare and balanced between groups. Two cases of infusion-site phlebitis were observed in the desmopressin arm (4.2%) and one case in the placebo arm (2.1%). No episodes of hyponatremia were documented during follow-up. No cases of anaphylaxis or clinically relevant allergic or skin reactions were observed.

## DISCUSSION

In this double-blind, randomized, placebo-controlled trial, we evaluated the efficacy and safety of prophylactic intravenous desmopressin administration before percutaneous kidney allograft biopsy in adult transplant recipients at high risk of bleeding. No statistically significant differences were observed between the desmopressin and placebo groups in the incidence of the primary outcome, procedure-related bleeding complications, or any of the predefined secondary endpoints, including major and minor bleeding events, or hyponatremia. These findings suggest that, in this population, routine pre-biopsy desmopressin use does not confer a measurable clinical benefit.

Importantly, although desmopressin was not associated with a reduction in bleeding risk, biopsy indication emerged as a strong and independent determinant of minor bleeding. Surveillance biopsies were associated with a markedly higher risk of bleeding compared with indication biopsies, a finding that remained robust across adjusted and sensitivity analyses. This observation provides clinically relevant insight into bleeding risk stratification among transplant recipients undergoing biopsy.

Our results are consistent with several previous randomized and observational studies that did not demonstrate a clear benefit of desmopressin in reducing post-biopsy bleeding, particularly in high-risk patients with impaired renal function [5, 6, 15–17]. While some earlier studies reported reductions in minor bleeding or hematoma formation [4, 7, 8], these effects have not consistently translated into reduced major bleeding rates or clinically meaningful improvements. By focusing exclusively on kidney allograft biopsies, a population underrepresented in the literature, and applying rigorous trial methodology, our study strengthens the evidence in a limited but clinically relevant field.

Bleeding following allograft kidney biopsy has implications beyond those observed in the general nephrology population. In transplant recipients, even a single episode of major hemorrhage may necessitate blood transfusion, which has potential consequences including alloimmune sensitization and increased risk of antibody-mediated rejection, threatening long-term graft survival [9, 10]. Accordingly, minimizing exposure to blood transfusion is particularly important in this population, given the well-established link between transfusion-related alloimmunization and adverse graft outcomes [9, 10]. Furthermore, severe bleeding events may lead to graft loss or require invasive interventions, such as embolization or nephrectomy. Even minor bleeding complications may prolong hospitalization, delay diagnostic workup, and affect patient morbidity. Therefore, strategies aimed at minimizing bleeding risk in this population have both short- and long-term clinical relevance.

Several factors may explain the absence of demonstrable benefit from desmopressin in our trial. First, biopsies were performed by experienced nephrologists using real-time ultrasound guidance and standardized protocols that likely minimized baseline bleeding risk [1, 2]. Second, strict eligibility criteria, such as exclusion of patients with coagulopathies or uncontrolled hypertension, ensured a clinically stable cohort, potentially reducing the relative benefit of additional pharmacologic intervention. Finally, although desmopressin promotes platelet adhesion, its hemostatic effect may not be sufficient to overcome mechanical or procedural contributors to bleeding in this context.

From a safety perspective, desmopressin was well tolerated. Adverse events were rare and comparable between groups, and no cases of hyponatremia were observed. This aligns with the established safety profile of desmopressin on short-term, single-dose use [18]. Nevertheless, the lack of efficacy raises questions about the routine use of desmopressin in this population, particularly given cost and resource considerations.

This study has limitations. First, while the study was adequately powered to detect a clinically relevant difference in overall bleeding, based on prior data, it was not powered to evaluate differences in major bleeding, given the very low expected incidence of this outcome in allograft biopsies. As a consequence, the CIs for major bleeding are wide, and the risk of type II error for rare outcomes is substantial. Second, the trial was conducted at a single quaternary transplant center with expert operators, which may limit generalizability to other settings with different technical expertise. Third, despite targeting a high-risk population (eGFR <60 ml/min/1.73 m²), our exclusion criteria (uncontrolled hypertension or significant coagulopathy) may have led to the enrollment of patients with a lower absolute risk of bleeding, reducing the likelihood of detecting an effect. Fourth, hematoma volume was not systematically assessed and therefore could not be analyzed. Fifth, subgroup and interaction analyses were limited by sample size within strata, and therefore should be interpreted cautiously. Finally, desmopressin was administered intravenously, a route chosen to ensure predictable bioavailability and rapid onset; however, alternative routes of administration, such as intranasal or subcutaneous delivery, were not evaluated and may have different pharmacokinetic or pharmacodynamic profiles.

In conclusion, prophylactic desmopressin administration prior to percutaneous kidney allograft biopsy did not reduce bleeding complications in adults at increased risk of bleeding. When biopsies are performed under optimal technical conditions with appropriate patient preparation, desmopressin does not appear to reduce the risk of bleeding complications. Surveillance biopsy indication emerged as a key determinant of bleeding risk and may help inform future strategies for patient selection and risk mitigation. These results reinforce the importance of focusing on procedural quality and individualized risk assessment over universal pharmacologic prophylaxis. Future studies should explore whether specific subgroups, such as patients with severe uremic burden or documented platelet dysfunction, could benefit from targeted desmopressin use, and whether risk prediction strategies incorporating biopsy indication can better guide preventive approaches.

## ETHICS APPROVAL AND CONSENT TO PARTICIPATE

The Hospital de Clínicas de Porto Alegre committee of research ethics granted ethical approval for this study. All participants provided written informed consent prior to enrollment.

## Data Availability

The data underlying this article are available in the article and in its online supplementary material.
